# Understanding of ironic and literal utterances in 4–9-year-old typically developing children: role of age, narrative skills, emotion comprehension and executive functions

**DOI:** 10.3389/fpsyg.2025.1718227

**Published:** 2025-12-17

**Authors:** Ekaterina Oshchepkova, Natalia Kartushina, Maria Kovyazina

**Affiliations:** 1 Faculty of Psychology, Lomonosov Moscow State University, Moscow, Russia; 2 Institute of Linguistics and Scandinavian Studies, University of Oslo, Oslo, Norway

**Keywords:** executive functions, irony, irony understanding, pragmatic development, preschool age, test of emotion comprehension

## Abstract

Language comprehension is essential to communication, yet understanding varies between literal meaning and verbal irony. While children grasp words' surface meanings, interpreting verbal irony requires them to consider context and infer the speaker's true intent, revealing a deeper layer of meaning. What factors contribute to this complex skill? The current study examined the impact of sex, narrative skills, executive functions (working memory and inhibitory control), theory of mind and emotion comprehension, while controlling for age, on understanding of literal statements and verbal irony in 267 typically developing 4–9-year old children (130 boys). To assess children's irony comprehension, we presented them with cartoon scenarios and asked to choose an emoji to indicate whether a parent's reaction was literal or ironic. A full-null model approach was used to examine the impact of children's cognitive, executive, emotional, and narrative skills on their irony understanding, where the full contained our factors of interest and the null contained only age and sex. The results indicated that age was the only factor related to literal meaning comprehension. In contrast, both age and working memory were significantly related to irony understanding. These findings can be used as a foundation for developing training to enhance non-literal language and pragmatic skills in children.

## Introduction

Language comprehension is a fundamental prerequisite for effective social interaction and communication. A particularly important aspect of language comprehension is the ability to grasp non-literal meaning, especially irony. Irony is defined as “the expression of one's meaning by using language that normally signifies the opposite, typically for humorous or emphatic effect” (OED: Oxford English Dictionary, 2025). Ironic statements, or verbal irony, are part of the broader construct of semantic and pragmatic skills. Irony (or verbal irony) involves a second-order meta-representation, meaning that in order to grasp it, the listener must (a) comprehend the utterance and (b) recognize the speaker's dismissive or distancing attitude toward the attributed thought (Köder and Falkum, 2021).

The question of when children begin to understand irony remains a matter of active research and debate. While some studies have found indications of irony understanding as early as ages 3–4 (Loukusa and Leinonen, 2008; Recchia et al., 2010), most research suggests that irony understanding typically emerges at ages 4–5, requiring additional cognitive effort and longer processing time compared to non-ironic utterances (Banasik-Jemielniak and Bokus, 2019). Massaro et al. (2013) conclude that 5-year-olds demonstrate a clear understanding of ironic intent. However, other researchers remain skeptical, showing that even 8-year-olds may struggle with the intended meaning of ironic statements (Nicholson et al., 2013). Some scholars argue for an even later developmental onset, pointing out that even 13-year-olds may not fully grasp implicatures (Demorest et al., 1984). Although the study of irony comprehension in children spans more than 40 years (Fuchs, 2023), the precise roles played by executive functions, Theory of Mind, and linguistic skills remain an open question (Fuchs, 2023). Given the lack of consensus on the developmental trajectory of irony comprehension, the present study aimed to address this question in Russian children aged 4–9.

Additionally, there is a lack of research examining to what extent are the comprehension processes for literal and non-literal (in particular, ironic) utterances similar or different in terms of the psychological characteristics of typically developing children that support them.

Among the most frequently discussed cognitive skills associated with children's processing of literal and non-literal meanings are executive functions, theory of mind, emotion comprehension, and narrative skills. The following sections outline the role of each factor in comprehension of literal and non-literal utterances.

## Factors contributing to comprehension of literal utterances in children

According to Fuchs (2023), key contributors to irony understanding in children include executive functions, theory of mind, emotional comprehension, and linguistic skills. It is unclear, however, whether the same factors are involved in understanding literal utterances.

Executive functions (EF) are mental processes that regulate many aspects of cognition and behavior (Miyake et al., 2000). One widely accepted framework for describing executive functions is that of Miyake et al. (2000), which identifies its key components such as cognitive flexibility, inhibitory control, and working memory (both visual and auditory). The role of executive functions in language comprehension can be understood as part of their overall contribution to language processing (Shokrkon and Nicoladis, 2022), supporting language comprehension (Ye and Zhou, 2009), with specific roles of working memory (Gathercole and Baddeley, 2014) and cognitive flexibility (Filipe et al., 2023).

The Theory of Mind (ToM) is understood as “the ability of an individual to make inferences about what others may be thinking or feeling and to predict what they may do in a given situation based on those inferences” (Schlinger, 2009, p. 435). It is related to language comprehension (Antonietti et al., 2006; Zufferey, 2010). Nevertheless, the impact of theory of mind on utterances comprehension, with the factor of age controlled for, has not yet been investigated.

Emotion comprehension (EC), following the approach of Pons and Harris, is defined as the ability to understand “the nature, causes, and consequences of the emotional experience in the self and others” (Pons and Harris, 2019, p. 431). Children's development of EC progresses through several stages (Pons and Harris, 2004, 2005): “External” level (ages 3–5), “Mental” level (ages 4–7), and “Reflective” level (ages 6–10). While the connection between children's EC (and emotional development in general) and language comprehension is well-documented (Beck et al., 2012; Conte et al., 2019), the specific contribution of EC to utterances comprehension has yet to be demonstrated.

Narrative skills are children's ability to construct coherent and cohesive stories based on a sequence of episodes. The narratives can be evaluated in terms of their macrostructure (overall organization and logical liens), microstructure (language specific correctness), and the use of internal state terms (lexical units denoting feelings, emotions, perception and other internal states) (Gagarina et al., 2012). While both narrative skills and utterance comprehension are components of language competence, however, it remains unclear which aspect of narrative skills—macrostructure, microstructure, or the use of internal state terms—contributes to utterance comprehension, and whether such a relationship exists at all.

Therefore, while separate pieces of evidence support a relationship between utterance comprehension and other cognitive domains (namely, executive functions, emotion comprehension, theory of mind, and narrative skills), the unique contribution of these domains to comprehension, after accounting for age, has not been investigated.

## Factors contributing to comprehension of non-literal utterances, in particular irony, in children

In this section we examine how the aforementioned cognitive abilities contribute to comprehension of non-literal (ironic) utterances.

In the domain of EF, inhibitory control has been found to play a significant role in irony comprehension (Caillies et al., 2014). As for the working memory, research with adults suggests that fluid intelligence, rather than working memory, modulates irony processing (Kyriacou and Köder, 2024). By contrast, Antoniou and Milaki (2021) showed that in bidialectal speakers, working memory substantially facilitates faster irony comprehension. Further studies with adults have likewise reported significant correlations between verbal working memory and the understanding of irony and sarcasm (Godbee and Porter, 2013). Yet, no study examined the role of executive functions on comprehension of ironic utterances in children with the factor of age controlled for.

Turning to the Theory of Mind (ToM), findings indicate that ToM significantly supports irony understanding in autistic children (Song et al., 2024), whereas no such effect has been observed in 5–8 year-old typically developing children (Szücs and Babarczy, 2017).

Evidence regarding the role of emotion understanding in comprehension of non-literal utterances remains mixed. Bajerski (2016), for example, found that in adults, emotional intelligence was negatively associated with irony understanding across several dimensions. In contrast, Nicholson et al. (2013) reported that for 8–9-year-old children, EC makes a substantial contribution to irony understanding.

Thus, although the general theoretical framework (Pexman, 2023) assumes that understanding irony requires linguistic knowledge, working memory, and cognitive flexibility, empirical research specifically on children aged 4–9 remains insufficient.

Another point of debate concerns the relative importance of contextual information vs. vocal intonation in children's irony understanding (Fuchs, 2023). Although considerable evidence advocates for the role of intonation as a key factor (Köder and Falkum, 2021), the issue is not yet settled (Fuchs, 2023). Regarding the comprehension of intonation, research indicates that working memory is a significant predictor of its development (Kuang et al., 2024; Stepanov et al., 2020).

## Research relevance and aim

Thus, the impact of EF, EC, ToM, and narrative skills on the comprehension of both literal and ironic utterances in children remains understudied. This research question is highly relevant not only for designing intervention programs for children with ASD and communication disorders but also for supporting the development of social-communicative skills in typically developing children. The aim of the present study is to assess and compare the contributions of EF, EC, ToM, and narrative skills to the comprehension of literal and ironic utterances, as key aspects of pragmatic competence, in children aged 4 to 9 years.

## Methods and sample

### Sample

The sample consisted of 267 monolingual Russian children aged 4 to 9 years (M = 82 months; SD = 17), of whom 130 were boys. Of these, 109 children attended senior or preparatory preschool groups (ages 51–74 months), while 158 were in the first or second grades of primary school (ages 81–111 months). All children came from families of middle socio-economic status. Children were tested in two sessions. Children who did not complete both testing sessions, as well as those whose parents did not provide written informed consent, were excluded from the study.

### Instruments

To assess children's comprehension of ironic and non-ironic utterances we used the tasks developed by Köder and Falkum (2021). The original tasks were used with Norwegian children aged 2 to 8 years, as well with adults. The tasks were translated and adapted for Russian-speaking children. These are 12 illustrated stories in which parents ask their children to perform or not certain actions (e.g., washing hands before eating, playing with a sibling, not soiling the floor). The children either comply with or ignore the request. Then, the parents provide feedback: either in a literal praise (e.g., “Good job, well done!”) or literal criticism (e.g., “That was very bad”). In three out of the 12 scenarios, the child fails to comply with the parent's request, and the parent uses ironic praise “Good job!”, implying criticism. The participants are instructed to choose either a “happy” or a “sad” smile to report the real meaning of a parent's feedback. Each correct answer earns the child one point, so a total score can range between 0 and 9 for the understanding of literal statements, and 0 and 3 for the irony-specific scenarios.

To assess EC, we used the TEC (Test of Emotion Comprehension) (Pons and Harris, 2004). TEC is a tool that allows to capture child emotion understanding and measures its nine components: (1) emotion recognition, (2) external cause, (3) desire, (4) belief, (5) reminder, (6) regulation, (7) hidden, (8) mixed, and (9) morally-based emotions. The test consists of 22 tasks. An accurate answer receives 1 point, and a wrong answer receives a zero. The total raw score is the sum of points for all 22 tasks, ranging from 0 to 22 points. The reliability of this instrument in Russian-speaking children has been demonstrated previously (Bukhalenkova et al., 2024).

To assess EF, the NEPSY-II assessment battery was used (Korkman et al., 2007). *The NEPSY battery was used because, on the one hand, it allows for the assessment of the relevant cognitive skills in children aged 3 to 16.11 (Davis and Matthews, 2010), and on the other hand, it has been adapted and validated for Russian-speaking children (Almazova et al., 2019). Research has also shown that this battery is suitable for children as young as 4 years (Veraksa et al., 2023)*. Using this instrument, the following components were assessed in children: inhibitory control and working memory (both visual and auditory). To assess inhibitory control, the Inhibition subtest (NEPSY-II) was used. Tasks performance correctness and time were assessed. Working memory was assessed through Sentence repetition (auditory working memory, maximum score-−34) and Memory for designs (visual working memory, maximum score-−120). The test had been translated into Russian, adapted for use with Russian-speaking children aged 5 to 8 years and showed high reliability (Veraksa et al., 2020).

To assess narrative skills, we used pictures from the MAIN (Multilingual assessment instrument for narratives) (Gagarina et al., 2012). The child was shown a sequence of six pictures bound into a booklet with three episodes. After examining the pictures, the child was instructed to tell what has happened there so it becomes a real story. Children's narratives were evaluated on the narrative's macrostructure: coherence with the narrative structure and the adequacy of the story (scored 2–20 points); and on the narrative's microstructure: lexical and grammatical quality of the narrative (scored 2–20 points) and the number of Internal State Terms (IST). Each IST earned the child one point, with no upper limit.

Theory of Mind (ToM) was assessed using a subtest of NEPSY-II, that included 21 emotion, social, false belief and second-order false belief tasks. The maximum score for the test was 28 points (several tasks give 2 or 3 points, all the other give 1 point). The test includes tasks on emotion recognition, false-belief tasks and tasks on metaphor comprehension.

### Procedure

The assessment was conducted by trained testers, individually with each child, in a quiet and bright room of the children's kindergarten. Two sessions were organized with each child, lasting 15–20 min each. Children were free to stop the test at any time. All methods were presented to the children in the same established order: at the first session, children performed the narratives, the TEC, and the Memory for Designs subtest; at the second session, they performed the Inhibition and the Sentence repetition subtests and the tasks for understanding of ironic and literal utterances. Prior to the study, the testers completed specialized training sessions on the administration and scoring procedures of the specified methods.

All the parents were informed about the study goals and gave written consent for their children's participation in the research. The study was carried out in those educational institutions with which cooperation agreements were concluded, and parental consent was collected with the help of the educators working in the groups attended by the children.

### Statistical analysis

We conducted all analyses using *R* (R Core Team, 2020). To examine the role of EF, EC, ToM, and narrative skills in children's comprehension of ironic and literal utterances, we used a full–null model comparison approach (a statistical method to compare two models to see if your main predictor variable is meaningfully improving the model's ability to explain the data), which minimizes type I errors by addressing the problem of multiple testing (Forstmeier and Schielzeth, 2011). For both literal and ironic utterances, we compared a model that included our predictors of interest with a respective null model that did not, using likelihood ratio tests. The null models contained only age and sex. The full models included, in addition, EF (visual working memory, auditory working memory, inhibitory control), EC (total score), ToM (total score), and narrative skills (macrostructure, microstructure, and use of internal state terms). The dependent variable was accuracy (1 = correct, 0 = incorrect) for each of the 9 non-ironic and 3 ironic scenarios per child. If the full–null comparison was significant, fixed effects were derived using the Anova function from the car package (Fox and Weisberg, 2018). *p*-values were obtained via the lmerTest package using Satterthwaite's approximation (Kuznetsova et al., 2017). Age was z-transformed.

## Results

### Role of executive functions, emotion understanding, and narrative skills on literal utterances understanding

The null model examining the impact of age and sex on accuracy of non-ironic statement understanding revealed a significant positive effect of age [β = 1.66, OR = 1.66, 95% CI [1.24, 2.22], *p* = 0.001]. Sex was not significant (OR = 1.48, *p* = 0.166).

The full-null model comparison showed that the full model did not provide a significantly better fit to the data than the simpler age-and-sex model, i.e., the null [χ^2^_(8)_ = 11.74, *p* = 0.163]. The Marginal *R*^2^ and Conditional *R*^2^ were 0.034 and 0.594, respectively, indicating that once age is accounted for, the individual differences in cognitive and language skills do not add in predicting variance in children's comprehension of literal language (see Table 1).

**Table 1:** Regression model of literal utterances understanding.

**Predictors**	**Accuracy**		
	**Odds ratios**	**CI**	***p***
(Intercept)	184.43	14.44–2355.92	< 0.001
*z* age	1.44	0.96–2.15	0.079
Sex [2]	1.45	0.81–2.61	0.212
Visual working memory	0.99	0.97–1.00	0.097
TEC	1.21	0.98–1.48	0.073
ToM	1.04	0.94–1.15	0.436
Narratives (macro)	0.92	0.82–1.03	0.163
Narratives (micro)	0.97	0.84–1.12	0.686
Inhib control	0.98	0.93–1.02	0.278
IST	1.08	0.93–1.25	0.337
Auditory working memory	1.05	0.96–1.15	0.284
**Random effects**			
σ^2^	3.29		
τ_00__ID_	0.46		
τ_00__irony_item_	4.13		
ICC	0.58		
N _ID_	266		
N_irony_item_	9		
Observations	2,394		
Marginal *R*^2^/conditional *R*^2^	0.063/0.609		

### Role of executive functions, emotion understanding, and narrative skills on ironic utterances comprehension

**Figure 1:**
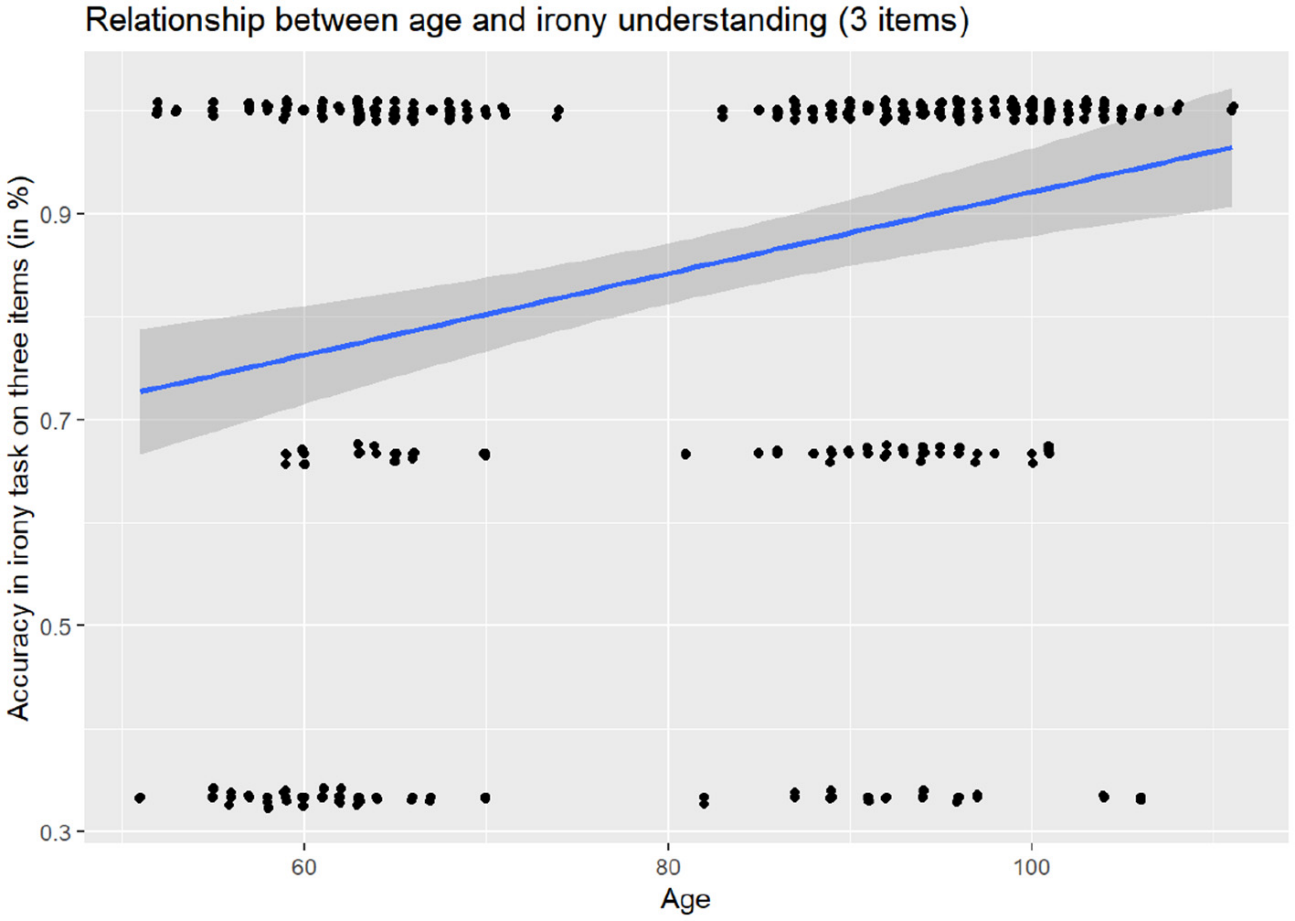
Relationship between accuracy in irony task understanding and age.

For the three irony-specific items, the null model showed a strong and significant effect of age [β = 4.52, OR = 4.52, 95% CI [2.03, 10.07], *p* < 0.001], see Figure 1. Sex was again non-significant (OR = 1.51, *p* = 0.477).

A full-null model comparison was marginally significant [χ^2^_(9)_ = 16.69, *p* = 0.054], indicating that including the target factors showed an improvement in the fit over the age-only model.

**Figure 2:**
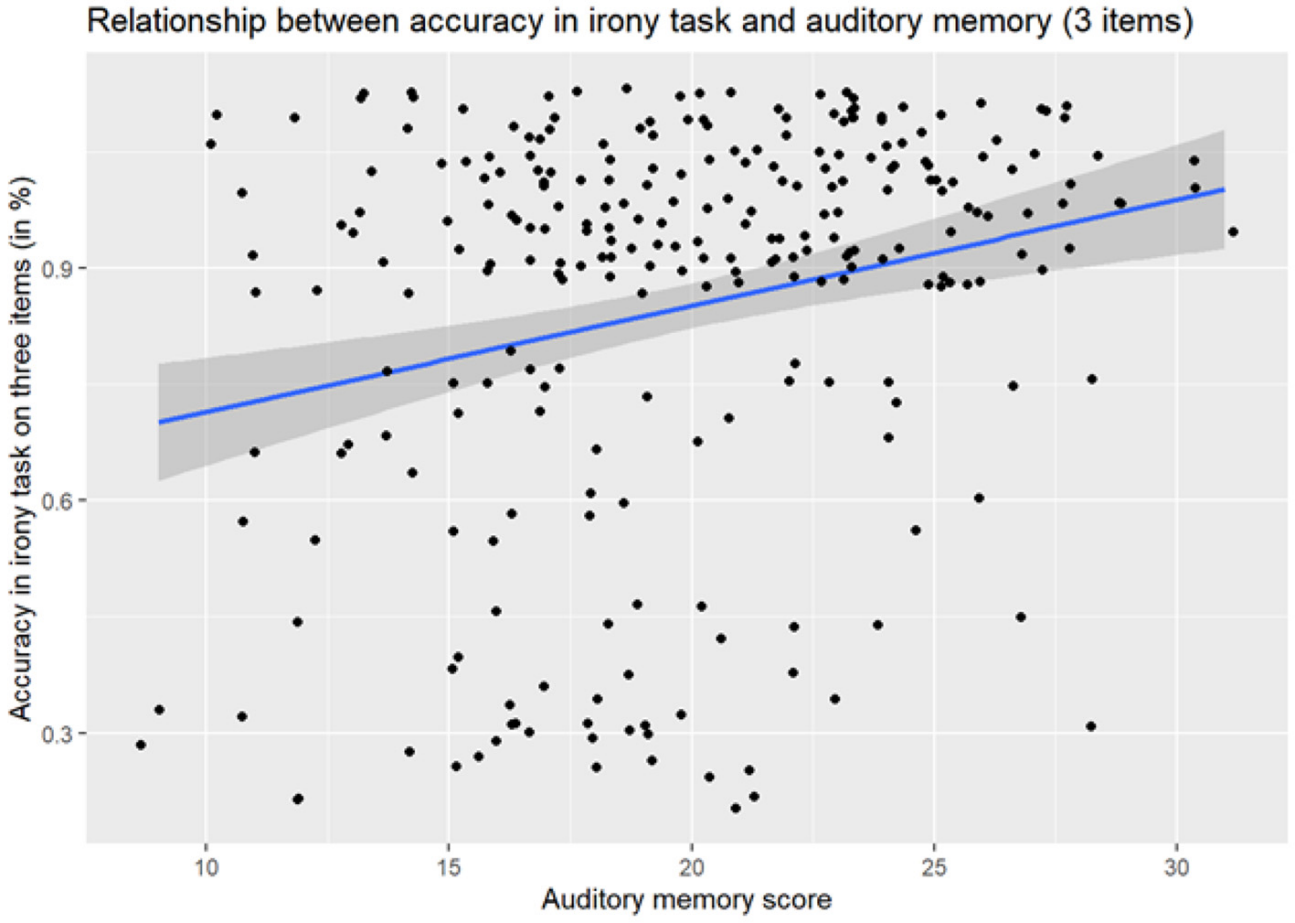
Relationship between accuracy in irony task understanding and verbal working memory.

A subsequent reduced model (after removing non-significant factors of p > 0.1 from the full model) revealed that the accuracy on ironic items was significantly predicted by: Age [OR = 2.24, 95% CI [1.02, 4.91], *p* = 0.044], internal state terms in narratives [OR = 1.38, 95% CI [1.01, 1.88], *p* = 0.041] and Auditory Working Memory [OR = 1.19, 95% CI [1.02, 1.40], *p* = 0.032] (see Figure 2).

**Figure 3:**
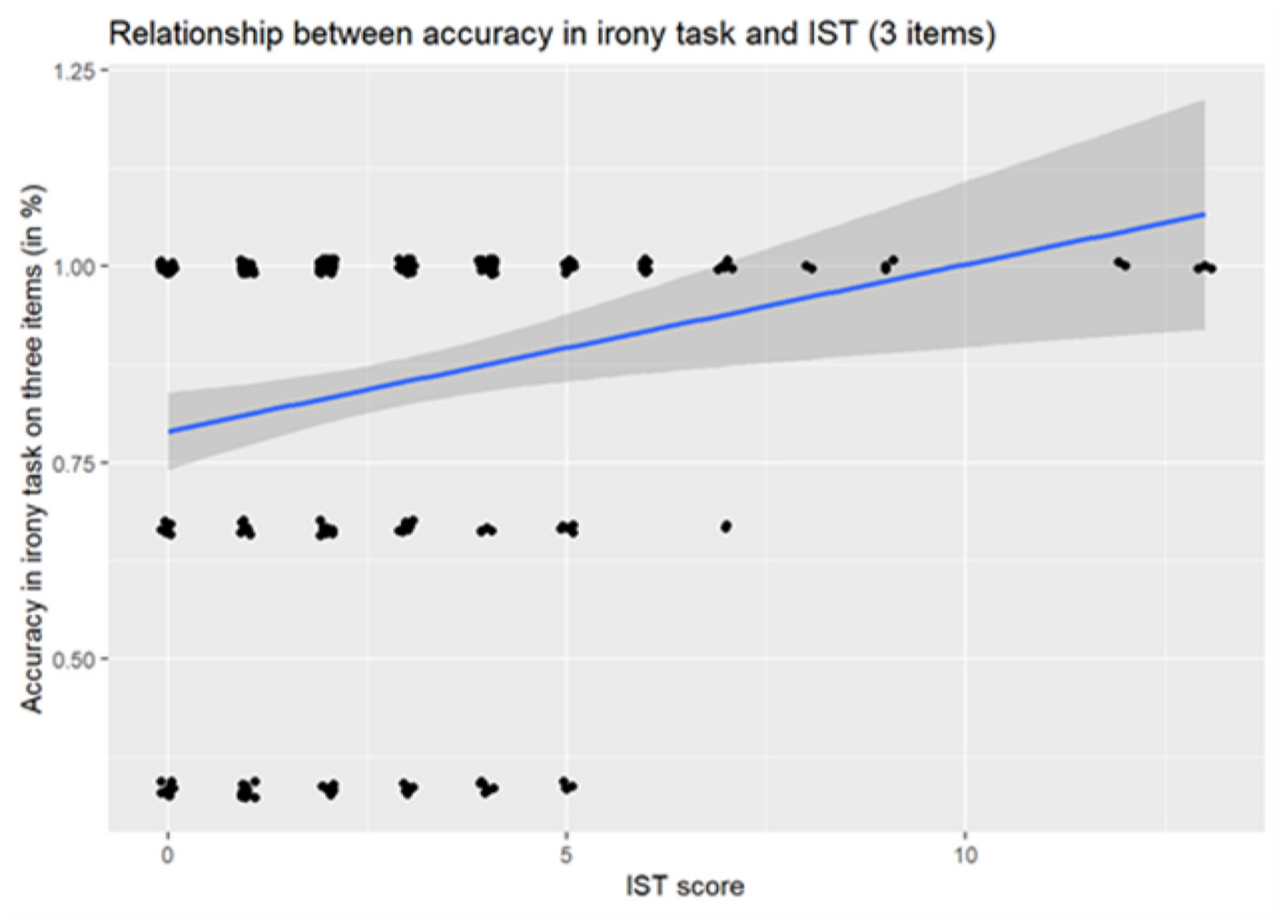
Relationship between accuracy in irony task understanding and usage of internal state terms in narration.

A visual inspection of the IST scores indicated that the effect of IST in narratives was driven by a small number of high-performing outliers (*n* = 9), see Figure 3. When these participants were removed, the effect of IST was no longer significant (OR = 1.28, *p* = 0.149). However, the effect of auditory working memory remained significant [OR = 1.20, 95% CI [1.02, 1.41], *p* = 0.029] alongside the effect of age.

## Discussion

The first of our two main research aims was to examine whether, factors such as EF, EC, ToM, and narrative skills contribute to the comprehension of literal utterances in typically developing 4–9-year-old children, when controlling for age. In a sample of 267 children, we found that when age is included in the analyses, none of these skills was related to literal comprehension. Thus, contrary to expectations based on previous research, neither EF, nor EC, nor ToM, nor even narrative skills contributed significantly to literal comprehension. This effect may be due to the fact that, in our study, a ceiling in the comprehension of literal utterances was reached quite early: by the age of five, almost all children were already giving correct answers to all tasks. Therefore, more complex situations may be needed to obtain similar results.

The second aim was to investigate whether, the same factors contribute to the comprehension of ironic utterances in 4–9-year-old, when controlling for age. In the same sample, we found that, in addition to age, only EF—specifically auditory working memory—made a stable contribution to variance in irony comprehension. This finding aligns with Baddeley's working memory theory (Baddeley, 2017), according to which irony comprehension can be explained as the construction of a mental (situational) model. The child's working memory holds key elements (subjects, actions, objects) and integrates them with each other. This allows the child to analyze the situation depicted and the parent's words, and on this basis to infer whether the utterance is literal or ironic. Children with higher auditory working memory capacity demonstrated stronger irony comprehension skills, suggesting that irony understanding might depend on children's ability to retain in working memory both the conversational context and the preceding utterance to which the ironic remark refers.

Verbal working memory has repeatedly been shown to contribute to the processing of intonational contours. Intonation, together with context, plays a central role in irony comprehension (Köder and Falkum, 2021). Thus, based on our findings, one may argue that intonation contributed most strongly in the present study, since if situational interpretation (depicted in the picture) had played a larger role, one would expect a greater contribution of visual working memory.

## Conclusion

Our study demonstrated a clear distinction in the contributions of cognitive and language skills to the comprehension of literal vs. ironic utterances in typically developing children. Specifically, while only age significantly predicted comprehension of literal utterances, both age and verbal working memory significantly predicted irony understanding. These findings may contribute to the development of educational and intervention programs aimed at enhancing children's understanding of non-literal meaning, thereby improving their working memory and executive functions in general.

A key limitation of our study is that, following Köder and Falkum's paradigm, we presented children with only 3 ironic utterances out of 12. As a result, the possible score range for irony comprehension was restricted (0–3), limiting variability. Regarding literal utterances, the task was too simple for the children, leading to a ceiling effect, which may also have constrained our findings. Future research needs to develop material that has more items to test irony comprehension (and across more situational contexts) that might potentially capture infants' variability in the task better.

## Data Availability

The raw data supporting the conclusions of this article will be made available by the authors, without undue reservation.
